# Square Root Convexity of Fisher Information along Heat Flow in Dimension Two

**DOI:** 10.3390/e25040558

**Published:** 2023-03-24

**Authors:** Junliang Liu, Xiaoshan Gao

**Affiliations:** KLMM, UCAS, Academy of Mathematics and Systems Science, Chinese Academy of Sciences, Beijing 100190, China

**Keywords:** Fisher information, heat equation, semidefinite programming, log-convex, square root convex, sum of squares

## Abstract

Recently, Ledoux, Nair, and Wang proved that the Fisher information along the heat flow is log-convex in dimension one, that is $\frac{d^{2}}{dt^{2}}\log\left(I\left(X_{t}\right)\right)\geq 0$ for n=1, where $X_{t}$ is a random variable with density function satisfying the heat equation. In this paper, we consider the high dimensional case and prove that the Fisher information is square root convex in dimension two, that is $\frac{d^{2}}{dt^{2}}\sqrt{I_{X}}\geq 0$ for n=2. The proof is based on the semidefinite programming approach.

## 1. Introduction

Let *X* be a random variable defined on $R^{n}$ with density function f(x), which is assumed to be differentiable. The *differential entropy* H(X) and the *Fisher information* I(X) of *X* are, respectively, defined to be

$$\begin{array}{ccc} & & H\left(X\right):=-\int_{R^{n}}f\left(x\right)\log f\left(x\right)dx\;\mathrm{and}\;I\left(X\right):=\int_{R^{n}}\frac{{\left|\nabla f\left(x\right)\right|}^{2}}{f(x)}dx. \end{array}$$

In 1948, Shannon [1] proposed the *entropy power inequality (EPI)* $N_{X+Y}\geq N_{X}+N_{Y}$, where *X* and *Y* are independent random variables defined by $R^{n}$ and $N\left(X\right):=\exp\left(\frac{2}{n}H\left(X\right)\right)/$(2πe). As one of the most important inequalities in information theory, Shannon’s EPI has many proofs and applications [2,3,4,5,6].

In 1985, Costa [7] proved a generalization of Shannon’s EPI, that is, the entropy power $N(X_{t})$ of $X_{t}=X+\sqrt{t}Z$ is concave in *t*, where *X* is a random variable and $Z=N(0,I_{n})$ is the *n*-dimensional standard normal distribution, independent of *X*. This inequality also has many proofs and applications [8,9,10,11].

Costa also proved that $\frac{d}{dt}H\left(X_{t}\right)\geq 0$ and $\frac{d^{2}}{dt^{2}}H\left(X_{t}\right)\leq 0$ [7] (Corollary 1). Along this line, Cheng and Geng [12] proposed the *completely monotone conjecture* (CMC)

$${\left(-1\right)}^{m+1}\frac{d^{m}}{dt^{m}}H\left(X_{t}\right)\geq 0,m\in N_{+}$$

and proved the conjecture for m=3,4 and n=1. Guo, Yuan, and Gao [13] proved the conjecture in the cases m=3,n=2,3,4 and the case m=4,n=2, using semidefinite programming (SDP) software programs. Other related results were also obtained based on the SDP approach [14,15].

The CMC was implicitly considered by Mckean [16] in studying the entropy for solutions of the heat equation $u_{t}=▵u$. The density function of $X_{t}$ is a solution of the heat equation $u_{t}=\frac{1}{2}▵u$ [2]. Interestingly, the converse is also true; that is, if the density function of a random variable $Y_{t}$ is a solution of the heat equation, then $Y_{t}$ has the form of $X_{t}$ [11]. Thus, studying properties of $H(X_{t})$ and $I(X_{t})$ are equivalent to studying that of a probability measure satisfying the heat equation.

Cheng and Geng [12] also proposed the *log-convexity conjecture*: the Fisher information along the heat flow is log-convex, which can be deduced from CMC. In 2021, Ledoux, Nair, and Wang [17] proved the log-convexity conjecture for n=1.

In this paper, we consider the two-dimensional case as suggested in [17]. We prove the *square root convexity* (abbr. sqrt-convexity) of Fisher information along heat flow in dimension two. Precisely, we prove the following result.

**Theorem** **1.** *Let X be a random variable defined on $R^{2}$, $Z=N(0,I_{2})$ a Gaussian variable independent of X, and $X_{t}=X+\sqrt{t}Z$. Then we have*

(1) $$\frac{d^{2}}{dt^{2}}\sqrt{I(X_{t})}\geq 0.$$

The main idea of the proof is that proof for inequality (1) can be reduced to the proof of whether a quadratic polynomial is a sum of squares (SOS) [18] of linear forms, which can be solved with SDP [19]. The SOS is explicitly given, which provides a rigourous proof for the theorem. The SDP problem related with Theorem 1 has 71 variables, which is difficult to solve by manual calculation.

We also show that log-convexity of the Fisher information along heat flow in dimension two cannot be proven with the SDP approach. More precisely, the SDP software program terminates, but fails to give a solution to prove the log-convexity. This does not imply that the log-convexity in dimension two is not correct, because the SOS problem to be solved with the SDP program is only a sufficient condition but not a necessary for the log-convexity. Theorem 1 is proven as a weaker form of the log-convexity conjecture for n=2. We also show that Theorem 1 implies the CMC for the third-order derivative in dimension two without assuming the log-concavity of p(x). Refer to Corollary 1 for details.

In Theorem 1, we do not assume that *X* is a log-concave variable. If adding the log-concave condition, then from Toscani [20], $\frac{1}{I(X_{t})}$ is concave, which implies inequality (1) and the proof can be found in Lemma 2.

**Figure 1:**
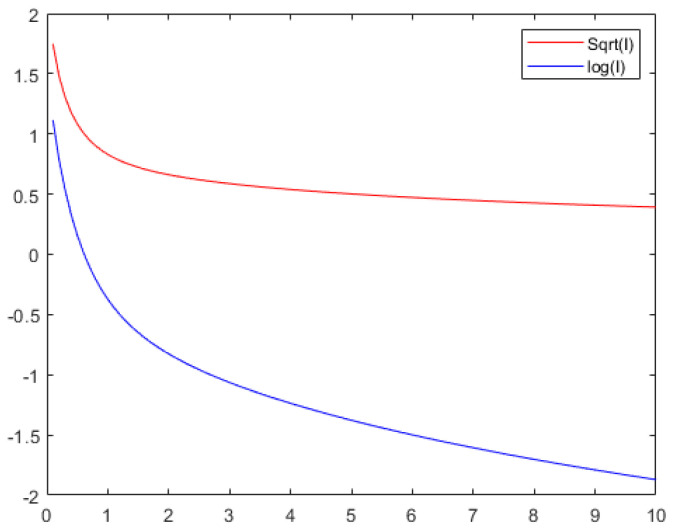
Figures for $\sqrt{I(X_{t})}$ and $\log I(X_{t})$ which are convex in *t*.

A drawback of the approach based on SDP is that the proof is difficult for people to check. Although the SOS gives an explicit proof for the theorem, it is quite large to be computed manually. To alleviate this problem, we give the programs and data in github.com, so that interested readers may check the proof using software systems. Refer to Remark 2 for details on how to do this. We also give an illustration for the method by proving Theorem 1 for the case *n* = 1 in Section 3.1. On the other hand, in the proof of information inequalities, it often happens that the computation is too large to be performed manually, and using computer programs becomes one of the major approaches in proving information inequalities [14,21,22,23,24,25]. To show our result more intuitively, we give the figures of $\sqrt{I(X_{t})}$ and $\log I(X_{t})$ in Figure 1, where $p(y_{1},y_{2})$ in Equation (2) is $\frac{y_{1}^{2}y_{2}^{2}}{2\pi }\exp\left(-\frac{y_{1}^{2}+y_{2}^{2}}{2}\right)$. In this case, both $\sqrt{I(X_{t})}$ and $\log I(X_{t})$ are convex in *t*.

## 2. Preliminaries

### 2.1. Notations and Preliminary Results

Let *X* be a random variable defined by $R^{n}$ with density function p(x), which is assumed to be differentiable and $Z=N(0,I_{n})$ the *n*-dimensional standard normal distribution, independent of *X*. Then $X_{t}=X+\sqrt{t}Z$ is also a random variable defined on $R^{n}$ with density function

(2) $$f\left(x,t\right):=\frac{1}{{\left(2\pi t\right)}^{n/2}}\int_{R^{n}}p\left(y\right)\exp\left(-\frac{{∥x-y∥}^{2}}{2t}\right)dy,$$

which is differentiable since p(x) is. It is known that f(x,t) satisfies the heat Equation (2)

$$\frac{\partial }{\partial t}f\left(x,t\right)=\frac{1}{2}▵f\left(x,t\right).$$

The *differential entropy* $H(X_{t})$ and *Fisher information* $I(X_{t})$ of $X_{t}$ are, respectively, defined as

$$\begin{array}{ccc} & & H(X_{t}):=-\int_{R^{n}}f\left(x,t\right)\log f\left(x,t\right)dx\;\mathrm{and}\;\\ & & I(X_{t}):=\int_{R^{n}}\frac{{\left|\nabla f\left(x,t\right)\right|}^{2}}{f(x,t)}dx. \end{array}$$

For convenience, we use H(t) and I(t) to denote $H(X_{t})$ and $I(X_{t})$ in the rest of the paper.

We can easily obtain the following relation between H(t) and I(t) by de Bruijn’s identity [2]:

(3) $$\frac{d}{dt}H\left(t\right)=\frac{1}{2}I\left(t\right).$$

By the definition of I(t), the Fisher information is always positive, so we can take the square root of it. By Equation (3) and the fact $\frac{\partial^{2}}{\partial t^{2}}H\left(t\right)\leq 0$ [7], the first derivative of the Fisher information is always negative:

(4) $$\frac{d}{dt}\sqrt{I(t)}=\frac{1}{2\sqrt{I(t)}}\frac{d}{dt}I\left(t\right)=\frac{1}{\sqrt{I(t)}}\frac{\partial^{2}}{\partial t^{2}}H\left(t\right)\leq 0.$$

A function f(t) is called *sqrt-convex* in *t* if the square root of f(t) is convex in *t*. The following lemma gives an equivalent form of sqrt-convexity, which will be used in the proof of Lemma 10.

**Lemma** **1.** *Theorem 1 is valid, that is, I(t) is sqrt-convex in t, if and only if*

(5) $$2I\left(t\right)\frac{d^{2}}{dt^{2}}I\left(t\right)-{\left(\frac{d}{dt}I\left(t\right)\right)}^{2}\geq 0.$$

**Proof.**  The convexity of $\sqrt{I(t)}$ is equivalent to the fact that second-order derivative of $\sqrt{I(t)}$ is positive. From Equation (4), we have

$$\begin{array}{cc} \frac{d^{2}}{dt^{2}}\sqrt{I(t)} & =-\frac{1}{4I\left(t\right)\sqrt{I(t)}}{\left(\frac{d}{dt}I\left(t\right)\right)}^{2}+\frac{1}{2\sqrt{I(t)}}\frac{d^{2}}{dt^{2}}I\left(t\right)\\ \; & =\frac{1}{4I\left(t\right)\sqrt{I(t)}}\left(2I\left(t\right)\frac{d^{2}}{dt^{2}}I\left(t\right)-{\left(\frac{d}{dt}I\left(t\right)\right)}^{2}\right). \end{array}$$

Since I(t)>0, the lemma is proven.    □

**Corollary** **1.** *If I(t) is sqrt-convex in t for n=2, then the CMC for the third-order with dimension two is correct.*

**Proof.**  Since $\frac{d}{dt}H\left(t\right)=\frac{1}{2}I\left(t\right)$, it suffices to prove $\frac{d^{2}}{dt^{2}}I\left(t\right)\geq 0$. Using Lemma 1, if I(t) is sqrt-convex in *t* for n=2, then we have $2I\left(t\right)\frac{d^{2}}{dt^{2}}I\left(t\right)\geq {\left(\frac{d}{dt}I\left(t\right)\right)}^{2}\geq 0$. Because I(t)>0, then $\frac{d^{2}}{dt^{2}}I\left(t\right)\geq 0$.    □

Lemma 2 gives the relationship among sqrt-convexity, log-convexity, and concavity of $\frac{1}{I(t)}$.

**Lemma** **2.** *If $\frac{1}{I(t)}$ is concave in t, then log(I(t)) is convex in t. If log(I(t)) is convex in t, then I(t) is sqrt-convex in t.*

**Proof.**  Since $\frac{d^{2}}{dt^{2}}\frac{1}{I(t)}=\frac{1}{I{\left(t\right)}^{3}}\left(2{\left(\frac{d}{dt}I\left(t\right)\right)}^{2}-I\left(t\right)\frac{d^{2}}{dt^{2}}I\left(t\right)\right)\leq 0$, we have $I\left(t\right)\frac{d^{2}}{dt^{2}}I\left(t\right)\geq 2{\left(\frac{d}{dt}I\left(t\right)\right)}^{2}\geq {\left(\frac{d}{dt}I\left(t\right)\right)}^{2}$. Then, $\frac{d^{2}}{dt^{2}}\log\left(I\left(t\right)\right)=\frac{1}{I{\left(t\right)}^{2}}\left(I\left(t\right)\frac{d^{2}}{dt^{2}}I\left(t\right)-{\left(\frac{d}{dt}I\left(t\right)\right)}^{2}\right)\geq 0$, which means that log(I(t)) is convex. Similarly, convexity of log(I(t)) means that $I\left(t\right)\frac{d^{2}}{dt^{2}}I\left(t\right)\geq {\left(\frac{d}{dt}I\left(t\right)\right)}^{2}$. Then we can obtain $2I\left(t\right)\frac{d^{2}}{dt^{2}}I\left(t\right)\geq {\left(\frac{d}{dt}I\left(t\right)\right)}^{2}$. By Lemma 1, I(t) is sqrt-convex in *t*.    □

We consider the two-dimensional case and suppose that the two variables are $x=\{x_{1},x_{2}\}$. For convenience, we use *f* instead of f(x,t) and $f_{a,b}$ instead of $\frac{\partial^{a+b}f\left(x,t\right)}{\partial x_{1}^{a}\partial x_{2}^{b}}$. Then we can rewrite the Fisher information as

(6) $$I\left(t\right)=\int_{R^{2}}\frac{f_{1,0}^{2}+f_{0,1}^{2}}{f}dx_{1}dx_{2}$$

and the heat equation as

(7) $$\frac{\partial f}{\partial t}=\frac{f_{2,0}+f_{0,2}}{2}.$$

By Equation (7), it is easy to see that for each $f_{a,b}=\frac{\partial^{a+b}f}{\partial x_{1}^{a}\partial x_{2}^{b}}$, we have

(8) $$\frac{\partial f_{a,b}}{\partial t}=\frac{\partial^{a+b}}{\partial x_{1}^{a}\partial x_{2}^{b}}\frac{\partial f}{\partial t}=\frac{\partial^{a+b}}{\partial x_{1}^{a}\partial x_{2}^{b}}\frac{f_{2,0}+f_{0,2}}{2}=\frac{f_{2+a,b}+f_{a,b+2}}{2}.$$

In the following, we formally define the concept of *differential forms*, which are used to reduce the size of the SDP problems to be solved. Refer to Remark 1 for details.

A *differential monomial* is of the form $M=\prod_{i=1}^{k}v_{i}^{n_{i}}$, where $v_{i}=f_{a_{i},b_{i}}$, $n_{i}\in N_{+}$, and $a_{i},b_{i}\in N$. We define the *order* of $v_{i}$ to be $\mathrm{ord}\left(v_{i}\right)=a_{i}+b_{i}$, the *total order* of *M* to be $\mathrm{ord}\left(M\right)=\sum_{i=1}^{k}n_{i}\cdot \mathrm{ord}\left(v_{i}\right)$. The *total degree* of *M* is $\deg\left(M\right)=\sum_{i=1}^{k}n_{i}$. A *differential polynomial* is a finite linear combination of differential monomials over Q. A differential polynomial *P* is called the *k-th order differentially homogenous polynomial*, or simply a *k-th order differential form*, if each of its differential monomial is of total degree *k* and total order *k*.

In Lemma 3, we compute the expression of $I\left(t\right),\frac{d}{dt}I\left(t\right),\frac{d^{2}}{dt^{2}}I\left(t\right)$.

**Lemma** **3.** *We have*

(9) $$\begin{array}{ccc} & & I\left(t\right)=\int_{R^{2}}\frac{I_{1}}{f}dx_{1}dx_{2},\;\frac{d}{dt}I\left(t\right)=\int_{R^{2}}\frac{I_{2}}{f^{3}}dx_{1}dx_{2},\;\frac{d^{2}}{dt^{2}}I\left(t\right)=\int_{R^{2}}\frac{I_{3}}{f^{5}}dx_{1}dx_{2}, \end{array}$$

*where each $I_{i}$ is a 2i-th order differential form for i=1,2,3.*

**Proof.**  By Equation (6),

(10) $$I_{1}=f_{1,0}^{2}+f_{0,1}^{2}$$

is a second-order differential form, so the lemma is correct for $I_{1}$. For $I_{2}$,

$$\frac{d}{dt}I\left(t\right)=\int_{R^{2}}\frac{1}{f}\frac{\partial I_{1}}{\partial t}-\frac{I_{1}}{f^{2}}\frac{\partial f}{\partial t}=\int_{R^{2}}\frac{1}{f^{3}}\left(f^{2}\frac{\partial I_{1}}{\partial t}-fI_{1}\frac{f_{2,0}+f_{0,2}}{2}\right).$$

Then,

(11) $$\begin{array}{cc} I_{2} & =f^{2}\frac{\partial I_{1}}{\partial t}-fI_{1}\frac{f_{2,0}+f_{0,2}}{2}\\ \; & =f^{2}f_{1,0}f_{3,0}+f^{2}f_{1,0}f_{1,2}+f^{2}f_{0,1}f_{2,1}+f^{2}f_{0,1}f_{0,3}\\ \; & \;-\frac{ff_{1,0}^{2}f_{2,0}}{2}-\frac{ff_{0,1}^{2}f_{2,0}}{2}-\frac{ff_{1,0}^{2}f_{0,2}}{2}-\frac{ff_{0,1}^{2}f_{0,2}}{2}\\ \; & =f_{1,0}\left(f^{2}f_{3,0}+f^{2}f_{1,2}-\frac{ff_{1,0}f_{2,0}}{2}-\frac{ff_{1,0}f_{0,2}}{2}\right)\\ \; & \;+f_{0,1}\left(f^{2}f_{2,1}+f^{2}f_{0,3}-\frac{ff_{0,1}f_{2,0}}{2}-\frac{ff_{0,1}f_{0,2}}{2}\right)\\ \; & =f_{1,0}F_{1,0}+f_{0,1}F_{0,1}, \end{array}$$

where

(12) $$\begin{array}{c} F_{1,0}:=f^{2}f_{3,0}+f^{2}f_{1,2}-\frac{ff_{1,0}f_{2,0}}{2}-\frac{ff_{1,0}f_{0,2}}{2},\\ F_{0,1}:=f^{2}f_{2,1}+f^{2}f_{0,3}-\frac{ff_{0,1}f_{2,0}}{2}-\frac{ff_{0,1}f_{0,2}}{2} \end{array}$$

are third-order differential forms.

Thus, $I_{2}$ is a fourth-order differential form. Similarly, we can show that $I_{3}$ is a sixth-order differential form:

(13) $$\begin{array}{cc} I_{3} & =f^{4}f_{3,0}f_{1,2}+f^{4}f_{2,1}f_{0,3}+f^{2}f_{2,0}f_{1,0}^{2}f_{0,2}+f^{2}f_{2,0}f_{0,1}^{2}f_{0,2}-f^{3}f_{3,0}f_{2,0}f_{1,0}\\ \; & \;-f^{3}f_{3,0}f_{0,2}f_{1,0}-f^{3}f_{2,1}f_{2,0}f_{0,1}-f^{3}f_{2,1}f_{0,2}f_{0,1}-f^{3}f_{1,2}f_{2,0}f_{1,0}-f^{3}f_{1,2}f_{0,2}f_{1,0}\\ \; & \;-f^{3}f_{0,3}f_{2,0}f_{0,1}-f^{3}f_{0,3}f_{0,2}f_{0,1}+1/2f^{2}f_{0,2}^{2}f_{0,1}^{2}+1/2f^{2}f_{2,0}^{2}f_{1,0}^{2}+1/2f^{2}f_{2,0}^{2}f_{0,1}^{2}\\ \; & \;+1/2f^{2}f_{0,2}^{2}f_{1,0}^{2}+1/2f^{4}f_{1,2}^{2}+1/2f^{4}f_{2,1}^{2}+1/2f^{4}f_{0,3}^{2}+1/2f^{4}f_{3,0}^{2}\\ \; & \;-1/4f^{3}f_{0,1}^{2}f_{4,0}+1/2f^{4}f_{0,1}f_{4,1}-1/4f^{3}f_{0,1}^{2}f_{0,4}-1/2f^{3}f_{0,1}^{2}f_{2,2}+f^{4}f_{0,1}f_{2,3}\\ \; & \;+f^{4}f_{1,0}f_{3,2}-1/2f^{3}f_{1,0}^{2}f_{2,2}-1/4f^{3}f_{1,0}^{2}f_{4,0}+1/2f^{4}f_{0,1}f_{0,5}-1/4f^{3}f_{1,0}^{2}f_{0,4}\\ \; & \;+1/2f^{4}f_{1,0}f_{5,0}+1/2f^{4}f_{1,0}f_{1,4}. \end{array}$$

The lemma is proven.    □

Inspired by Cauchy–Schwarz inequality, we obtain the following inequality which is used in the proof of Lemma 9.

**Lemma** **4.** *For functions $f_{1},f_{2},g_{1},g_{2}$ in $x=\{x_{1},x_{2}\}$, we have*

(14) $${\left(\int_{R^{2}}\left(f_{1}g_{1}+f_{2}g_{2}\right)dx_{1}dx_{2}\right)}^{2}\leq \int_{R^{2}}\left(f_{1}^{2}+f_{2}^{2}\right)dx_{1}dx_{2}\int_{R^{2}}\left(g_{1}^{2}+g_{2}^{2}\right)dx_{1}dx_{2}.$$

**Proof.**  Using the Cauchy–Schwarz inequality, we have $|f_{1}g_{1}+f_{2}g_{2}|\leq \sqrt{\left(f_{1}^{2}+f_{2}^{2}\right)\left(g_{1}^{2}+g_{2}^{2}\right)}$. Using the Cauchy–Schwarz inequality of integral form, we have

$$\begin{array}{c} {\left(\int_{R^{2}}\sqrt{\left(f_{1}^{2}+f_{2}^{2}\right)\left(g_{1}^{2}+g_{2}^{2}\right)}dx_{1}dx_{2}\right)}^{2}\leq \int_{R^{2}}\left(f_{1}^{2}+f_{2}^{2}\right)dx_{1}dx_{2}\int_{R^{2}}\left(g_{1}^{2}+g_{2}^{2}\right)dx_{1}dx_{2}. \end{array}$$

Combining the above two inequalities, we prove the lemma.    □

### 2.2. Constraints

The density function *f* and its derivatives satisfy certain integral equations, from which the constraints of the SDP problems to be solved are obtained. Due to these reasons, these integral equations are called constraints. Precisely, a 2m-th order differential form *R* is called a *2m-th order constraint*, if

$$\begin{array}{c} \int_{R^{2}}\frac{R}{f^{2m-1}}dx_{1}dx_{2}=0. \end{array}$$

It is easy to see that the equations in (9) are still valid if $I_{k}$ is replaced by $I_{k}+C_{k}$, when $C_{k}$ is a 2k-th order constraint. Guo, Yuan, and Gao [13] proposed a method to compute the constraints, which will be used here to compute the constraints in dimension two. In the following, we show how to compute the 2m-order constraints.

**Lemma** **5** ([13])**.** *Let $k,m_{i},n_{i}\in N_{+}$ and $f^{\left(m_{i}\right)}$ be the $m_{i}$-th order derivative of f in Equation (2). Then*

(15) $$\int_{-\infty }^{\infty }f\left[\prod_{i=1}^{k}\frac{{\left[f^{\left(m_{i}\right)}\right]}^{k_{i}}}{f^{k_{i}}}\right]{|}_{x_{a}=-\infty }^{\infty }dx_{b}=0,$$

*where a=1,b=2 or a=2,b=1.*

This lemma guarantees that when using the integration by parts, the integral term of lower dimensions vanishes. The following lemma shows how to generate constraints. We repeat the proof here, because the proof procedure will be used in the proof of Lemma 7.

**Lemma** **6.** *Let M be a differential monomial with total order 2m−1. Then we can use integration by parts to obtain a 2m-th order constraint from M.*

**Proof.**  Let $x_{a}$ be one of the variables $x_{1},x_{2}$, and $x_{b}$ be another variable. Then we have

$$\int_{R^{2}}\frac{\partial }{\partial x_{a}}\frac{M}{f^{2m-2}}dx_{a}dx_{b}=\int_{-\infty }^{\infty }f\frac{M}{f^{2m-1}}{|}_{x_{a}=-\infty }^{\infty }dx_{b}\overset{\left(15\right)}{=}0.$$

Then using integration by parts, we have

$$\begin{array}{cc} \int_{R^{2}}\frac{\partial }{\partial x_{a}}\frac{M}{f^{2m-2}}dx_{a}dx_{b}\; & =\int_{R^{2}}\frac{1}{f^{2m-2}}\frac{\partial M}{\partial x_{a}}-\left(2m-2\right)M\frac{\partial f}{\partial x_{a}}\frac{1}{f^{2m-1}}dx_{a}dx_{b}\\ \; & =\int_{R^{2}}\frac{1}{f^{2m-1}}\left(f\frac{\partial M}{\partial x_{a}}-\left(2m-2\right)M\frac{\partial f}{\partial x_{a}}\right)dx_{a}dx_{b}=0. \end{array}$$

Thus, $M^{\prime }:=f\frac{\partial M}{\partial x_{a}}-\left(2m-2\right)M\frac{\partial f}{\partial x_{a}}$ is a 2m-th order constraint and the lemma is proven.    □

## 3. Proof of Theorem 1

The proof of Theorem 1 mainly consists of two steps. The first step, summarized in Lemma 10, is used to reduce the proof of Theorem 1 to the proof of the non-negativeness for a quadratic form with undetermined coefficients. This step is given in Section 3.2, Section 3.3 and Section 3.4. The reduction has three main ingredients: (1) Constraints given in Lemma 8 are used to form the SOS and Lemmas 5 and 6 show how to compute the constraints. (2) Lemma 7 is used to reduce all involved quantities into quadratic forms in certain variables. (3) By introducing ${\tilde{J}}_{3}$ in Lemma 9 and using the Cauchy–Schwarz inequality in Lemma 4, the quantity ${\left(\int_{R^{2}}\frac{I_{2}}{f^{3}}dx_{1}dx_{2}\right)}^{2}$ is relaxed to a simple form.

The second step, given in Section 3.5, is to compute the undetermined coefficients of the quadratic form using SDP, which is summarized as Problem 1. This step has two sub-steps: (1) In Problem 2, the undetermined coefficients $\alpha_{i}$ and $\beta_{j}$ are computed by omitting the second degree terms. (2) In Problem 5, the undetermined coefficients $\lambda_{k}$ are computed using the values of $\alpha_{i}$ and $\beta_{j}$ obtained in the first sub-step. In these two sub-steps, the quadratic forms are linear in the undetermined coefficients which can be computed with SDP and the computation procedure is given in Problems 3 and 4.

### 3.1. An Illustrative Example

In this subsection, we will prove Theorem 1 for *n* =1 and use this as an illustration of our proving method.

By Lemma 1, it suffices to prove (5). For convenience, we write f(x,t) as *f* and $\frac{\partial^{a}}{\partial x^{a}}f\left(x,t\right)$ as $f_{a}$. Using Lemma 6, we can obtain the constraints ${\hat{E}}_{i},i=1,\ldots ,6$:

(16) $$\begin{array}{cc} \int_{R}\frac{3ff_{1}^{2}f_{2}-2f_{1}^{4}}{f^{3}}dx=0.\\ \int_{R}\frac{{\hat{E}}_{i}}{f^{5}}dx=0\left(i=1\ldots 6\right),\\ {\hat{E}}_{1}=f^{4}f_{1}f_{5}+f^{4}f_{2}f_{4}-f^{3}f_{1}^{2}f_{4},\; & {\hat{E}}_{2}=f^{4}f_{2}f_{4}+f^{4}f_{3}^{2}-f^{3}f_{1}f_{2}f_{3},\\ {\hat{E}}_{3}=f^{3}f_{1}^{2}f_{4}+2f^{3}f_{1}f_{2}f_{3}-2f^{2}f_{1}^{3}f_{3},\; & {\hat{E}}_{4}=2f^{3}f_{1}f_{2}f_{3}+f^{3}f_{2}^{3}-2f^{2}f_{1}^{2}f_{2}^{2},\\ {\hat{E}}_{5}=f^{2}f_{1}^{3}f_{3}+3f^{2}f_{1}^{2}f_{2}^{2}-3ff_{1}^{4}f_{2},\; & {\hat{E}}_{6}=5ff_{1}^{4}f_{2}-4f_{1}^{6}. \end{array}$$

By Lemma 3, we have

$$\begin{array}{cc} I(t) & =\int_{R}\frac{f_{1}^{2}}{f}dx,\\ \frac{d}{dt}I\left(t\right) & =\int_{R}\frac{2f^{2}f_{1}f_{3}-ff_{1}^{2}f_{2}}{2f^{3}}dx=\int_{R}\frac{f_{1}\left(2f^{2}f_{3}-ff_{1}f_{2}\right)}{2f^{3}}dx,\\ \; & =\int_{R}\frac{f_{1}\left(2f^{2}f_{3}-ff_{1}f_{2}+\alpha \left(3ff_{1}f_{2}-2f_{1}^{3}\right)\right)}{2f^{3}}dx,\\ \frac{d^{2}}{dt^{2}}I\left(t\right) & =\int_{R}\frac{2f^{4}f_{3}^{2}-4f^{3}f_{1}f_{2}f_{3}+2f^{4}f_{1}f_{5}+2f^{2}f_{1}^{2}f_{2}^{2}-f^{3}f_{1}^{2}f_{4}}{4f^{5}}dx\\ \; & =\int_{R}\frac{E_{2}}{4f^{5}}dx, \end{array}$$

where $E_{2}:=2f^{4}f_{3}^{2}-4f^{3}f_{1}f_{2}f_{3}+2f^{4}f_{1}f_{5}+2f^{2}f_{1}^{2}f_{2}^{2}-f^{3}f_{1}^{2}f_{4}$. By Lemma 4,

$$\begin{array}{cc} {\left(\frac{d}{dt}I\left(t\right)\right)}^{2} & \leq \int_{R}\frac{f_{1}^{2}}{f}dx\int_{R}\frac{{\left(2f^{2}f_{3}-ff_{1}f_{2}+\alpha \left(3ff_{1}f_{2}-2f_{1}^{3}\right)\right)}^{2}}{4f^{5}}dx,\\ \; & =I\left(t\right)\int_{R}\frac{E_{1}{\left(\alpha \right)}^{2}}{4f^{5}}dx, \end{array}$$

where $E_{1}\left(\alpha \right):=2f^{2}f_{3}-ff_{1}f_{2}+\alpha \left(3ff_{1}f_{2}-2f_{1}^{3}\right)$.

By Lemma 1, it suffices to find an α such that $2E_{2}-E_{1}{\left(\alpha \right)}^{2}\geq 0$ is true under the constraints ${\hat{E}}_{i},i=1,\ldots ,6$, which is a consequence of the following SOS:

(17) $$2E_{2}-E_{1}{\left(-\frac{1}{3}\right)}^{2}-4{\hat{E}}_{1}+4{\hat{E}}_{2}-2{\hat{E}}_{3}+\frac{4}{3}{\hat{E}}_{5}-\frac{4}{15}{\hat{E}}_{6}={\left(2f^{2}f_{3}-2ff_{1}f_{2}+\frac{2}{3}f_{1}^{3}\right)}^{2}+\frac{8}{45}f_{1}^{6}\geq 0.$$

By (16) and (17),

$$\begin{array}{cc} 2I\left(t\right)\frac{d^{2}}{dt^{2}}I\left(t\right)-{\left(\frac{d}{dt}I\left(t\right)\right)}^{2}\; & \geq I\left(t\right)\int_{R}\frac{2E_{2}-E_{1}{\left(\alpha \right)}^{2}}{4f^{5}}dx\\ \; & =I\left(t\right)\int_{R}\frac{2E_{2}-E_{1}{\left(-\frac{1}{3}\right)}^{2}-4{\hat{E}}_{1}+4{\hat{E}}_{2}-2{\hat{E}}_{3}+\frac{4}{3}{\hat{E}}_{5}-\frac{4}{15}{\hat{E}}_{6}}{4f^{5}}dx\\ \; & \geq 0. \end{array}$$

Theorem 1 of case *n* = 1 is proven.

Equation (17) can be obtained in two steps. In the first step, we compute α. Instead of $2E_{2}-E_{1}{\left(\alpha \right)}^{2}\geq 0$, we consider $2E_{2}-\left({\left(2f^{2}f_{3}-ff_{1}f_{2}\right)}^{2}+2\alpha \left(2f^{2}f_{3}-ff_{1}f_{2}\right)\left(3ff_{1}f_{2}-2f_{1}^{3}\right)\right)\geq 0$ under the constraints, which can be solved by SDP since α is linear in the expression. Suppose that the solution for α is $\alpha_{0}$.

In the second step, we check whether $2E_{2}-E_{1}{\left(\alpha_{0}\right)}^{2}\geq 0$ is valid under the constraints using SDP, and the SOS in (17) can be found. Details of the proof procedure are given in the rest of this section.

### 3.2. Compute Constraints

In this section, we compute the fourth-order and sixth-order constraints using Lemma 6. For instance, from the differential monomial $M=ff_{0,1}f_{2,0}$ with total order 3, we obtain two fourth-order constraints:

$$\begin{array}{cc} \; & C_{1}=f\frac{\partial M}{\partial x_{1}}-2M\frac{\partial f}{\partial x_{1}}=f^{2}f_{3,0}f_{0,1}+f^{2}f_{2,0}f_{1,1}-ff_{2,0}f_{1,0}f_{0,1},\\ \; & C_{2}=f\frac{\partial M}{\partial x_{2}}-2M\frac{\partial f}{\partial x_{2}}=f^{2}f_{2,1}f_{0,1}+f^{2}f_{2,0}f_{0,2}-ff_{2,0}f_{0,1}^{2}. \end{array}$$

By considering all differential monomials with total order 3 and total degree 3, we obtain 20 constraints. Some of the constraints cannot be divided by $f_{0,1}$ or $f_{1,0}$, which are not needed in the proof due to the form of $I_{2}$ in Equation (11). Finally, we obtain eight fourth-order constraints $f_{1,0}P_{i}\left(1\leq i\leq 4\right)$ and $f_{0,1}Q_{i}\left(1\leq i\leq 4\right)$, where

(18) $$\begin{array}{cc} P_{1}=3ff_{1,0}f_{2,0}-2f_{1,0}^{3},\; & P_{2}=3ff_{1,0}f_{1,1}-2f_{0,1}f_{1,0}^{2},\\ P_{3}=2ff_{0,1}f_{1,1}+ff_{0,2}f_{1,0}-2f_{0,1}^{2}f_{1,0},\; & P_{4}=2ff_{0,1}f_{2,0}+ff_{1,0}f_{1,1}-2f_{0,1}f_{1,0}^{2},\\ Q_{1}=3ff_{0,1}f_{0,2}-2f_{0,1}^{3},\; & Q_{2}=3ff_{0,1}f_{1,1}-2f_{0,1}^{2}f_{1,0},\\ Q_{3}=ff_{0,1}f_{1,1}+2ff_{0,2}f_{1,0}-2f_{0,1}^{2}f_{1,0},\; & Q_{4}=ff_{0,1}f_{2,0}+2ff_{1,0}f_{1,1}-2f_{0,1}f_{1,0}^{2}. \end{array}$$

Similarly, we obtain 136 sixth-order constraints $R_{j}\;\left(1\leq j\leq 136\right)$. In summary, we obtain constraints $f_{1,0}P_{i}\left(1\leq i\leq 4\right),f_{0,1}Q_{i}\left(1\leq i\leq 4\right)$, and $R_{j}\left(1\leq j\leq 136\right)$, which satisfy

(19) $$\begin{array}{c} \begin{array}{c} \int_{R^{2}}\frac{f_{1,0}P_{i}}{f^{3}}dx_{1}dx_{2}=\int_{R^{2}}\frac{f_{0,1}Q_{i}}{f^{3}}dx_{1}dx_{2}=0,i=1,\ldots ,4,\\ \int_{R^{2}}\frac{R_{j}}{f^{5}}dx_{1}dx_{2}=0,j=1,\ldots ,136. \end{array} \end{array}$$

### 3.3. Reduce to Quadratic Form

In order to obtain an SDP problem with a smaller size, we will reduce all differential polynomials in the proof into quadratic forms in a set of new variables $M=\{M_{i}\;:\;1\leq i\leq 14\}$ which are all the differential monomials with total order 3 and total degree 3:

(20) $$M=\left\{\begin{array}{cccc} M_{1}=f^{2}f_{3,0}, & M_{2}=f^{2}f_{2,1}, & M_{3}=f^{2}f_{1,2}, & M_{4}=f^{2}f_{0,3},\\ M_{5}=ff_{2,0}f_{1,0}, & M_{6}=ff_{2,0}f_{0,1}, & M_{7}=ff_{1,1}f_{1,0} & M_{8}=ff_{1,1}f_{0,1},\\ M_{9}=ff_{0,2}f_{1,0}, & M_{10}=ff_{0,2}f_{0,1} & M_{11}=f_{1,0}^{3}, & M_{12}=f_{1,0}^{2}f_{0,1},\\ M_{13}=f_{1,0}f_{0,1}^{2}, & M_{14}=f_{0,1}^{3}. \end{array}\right\}$$

We rewrite $F_{1,0},F_{0,1}$ in Equation (12) and $P_{i}\left(1\leq i\leq 4\right),Q_{i}\left(1\leq i\leq 4\right)$ in Equation (18) as linear forms in M:

(21) $$\begin{array}{cc} {\tilde{F}}_{1,0}=M_{1}+M_{3}-\frac{1}{2}M_{5}-\frac{1}{2}M_{9},\; & {\tilde{F}}_{0,1}=M_{2}+M_{4}-\frac{1}{2}M_{6}-\frac{1}{2}M_{10},\\ {\tilde{P}}_{1}=3M_{5}-2M_{11},\; & {\tilde{P}}_{2}=3M_{7}-2M_{12},\\ {\tilde{P}}_{3}=2M_{8}+M_{9}-2M_{13},\; & {\tilde{P}}_{4}=2M_{6}+M_{7}-2M_{12},\\ {\tilde{Q}}_{1}=3M_{10}-2M_{14},\; & {\tilde{Q}}_{2}=3M_{8}-2M_{13},\\ {\tilde{Q}}_{3}=M_{8}+2M_{9}-2M_{13},\; & {\tilde{Q}}_{4}=M_{10}+2M_{7}-2M_{12}. \end{array}$$

The following lemma shows that any sixth-order constraint can be reduced to another sixth-order constraint which can be written as a quadratic form in M.

**Lemma** **7.** *For any differential monomial M with total order 6 and total degree 6, we can compute a sixth-order differential form P such that*

$$\int_{R^{2}}\frac{M}{f^{5}}dx_{1}dx_{2}=\int_{R^{2}}\frac{P}{f^{5}}dx_{1}dx_{2}$$

*and P is a quadratic form in M in Equation (20).*

**Proof.**  Since *M* is a differential monomial with total degree 6 and total order 6, let $M=\prod_{i=1}^{6}v_{i}$ with $v_{i}=f_{a_{i},b_{i}}=\frac{\partial^{c_{i}}f}{\partial x_{1}^{a_{i}}\partial x_{2}^{b_{i}}}$ satisfying $c_{i}=a_{i}+b_{i}$, $\sum_{i=1}^{6}c_{i}=6$, and $c_{s}\geq c_{k}$ for s≤k. We call $\left(c_{1},\ldots ,c_{6}\right)$ the *order type* and $c_{1}$ the *leading order* of *M*.

If $c_{1}\geq 4$, similar to the proof of Lemma 6, we can use integration by parts to obtain a new polynomial $P_{1}$ with leading order $c_{1}-1$.

(22) $$\begin{array}{ccc} \int_{R^{2}}\frac{M}{f^{5}}dx_{1}dx_{2}\; & = & \int_{R^{2}}\frac{1}{f^{5}}\frac{\partial^{c_{1}}f}{\partial x_{1}^{a_{1}}\partial x_{2}^{b_{1}}}\prod_{i=2}^{6}v_{i}\;dx_{1}dx_{2}\\ \; & = & -\int_{R^{2}}\frac{\partial^{c_{1}-1}f}{\partial x_{1}^{a_{1}-1}\partial x_{2}^{b_{1}}}\frac{\partial }{\partial x_{1}}\left(\frac{1}{f^{5}}\prod_{i=2}^{6}v_{i}\right)\;dx_{1}dx_{2}, \end{array}$$

where we assume $a_{1}\geq 1$, without loss of generality. Let $P_{1}=f^{5}\left(\frac{\partial^{c_{1}-1}f}{\partial x_{1}^{a_{1}-1}\partial x_{2}^{b_{1}}}\frac{\partial }{\partial x_{1}}\left(\frac{1}{f^{5}}\prod_{i=2}^{6}v_{i}\right)\right)$. It is easy to see that $P_{1}$ is a sixth-order differential form. Since $c_{1}\geq 4$, we have $c_{i}\leq 2$ for i=2,…,6, and hence the leading orders of all monomials of $P_{1}$ are equal to or less than $c_{1}-1$. If the leading order of a monomial $\tilde{M}$ of $P_{1}$ is still equal to or more than 4, we can repeat procedure (22) for $\tilde{M}$ until the leading orders of all monomials of $P_{1}$ are equal to or less than 3.

After the above procedure, we obtain a sixth-order differential form $P_{1}$ such that the leading orders of all monomials of $P_{1}$ are equal to or less than 3. If the order type of a monomial $\tilde{M}$ of $P_{1}$ is (2,2,2,0,0,0), then we use procedure (22) to change $\tilde{M}$ to a differential polynomial $P_{2}$. It is clear that the leading orders of all monomials of $P_{2}$ are equal to or less than 3 and the order types of all monomials of $P_{2}$ are not (2,2,2,0,0,0). Using the above procedure, we may eliminate all monomials with order type (2,2,2,0,0,0). For instance, for the monomial $f^{3}f_{2,0}f_{1,1}f_{0,2}$ with order type (2,2,2,0,0,0), we can obtain a sixth-order differential form $f^{3}f_{2,1}f_{0,2}f_{1,0}+f^{3}f_{1,2}f_{1,1}f_{1,0}-2f^{2}f_{1,1}f_{0,2}f_{1,0}^{2}$.

After the above two reduction procedures, we obtain a differential polynomial *P* such that the leading orders of all monomials of *P* are equal to or less than 3 and the order types of all monomials of *P* are not (2,2,2,0,0,0). Then the order types of the monomials of *P* are

$$\begin{array}{c} (3,3,0,0,0,0),(3,2,1,0,0,0),(3,1,1,1,0,0),(2,2,1,1,0,0),(2,1,1,1,1,0). \end{array}$$

All monomials with the above order types can be written as $M_{i}M_{j}$ for certain $M_{i},M_{j}$ in Equation (20). For instance, the monomial $f^{4}f_{3,0}f_{2,1}$ has order type (3,3,0,0,0,0), which can be written as $M_{1}M_{2}$. Thus, *P* is a quadratic form in variables M. The lemma is proven.    □

Using Lemma 7 to each monomial of $I_{3}$ in Equation (13), we obtain a quadratic form ${\tilde{I}}_{3}$ in M

(23) $$\begin{array}{cc} {\tilde{I}}_{3} & =1/2M_{1}^{2}-M_{1}M_{5}+3/2M_{2}^{2}-3M_{2}M_{6}+3/2M_{3}^{2}+1/2M_{4}^{2}-2M_{4}M_{6}-M_{4}M_{7}\\ \; & \;-M_{4}M_{10}-1/2M_{5}^{2}+3/2M_{6}^{2}-3M_{7}^{2}-2M_{7}M_{10}+3M_{8}^{2}-5/2M_{9}^{2}-3/2M_{9}M_{11}\\ \; & \;+21M_{9}M_{13}-1/2M_{10}^{2}+3/5M_{11}^{2}+3M_{12}^{2}-15M_{13}^{2}+3/5M_{14}^{2} \end{array}$$

which satisfies

(24) $$\begin{array}{c} \int_{R^{2}}\frac{I_{3}}{f^{5}}dx_{1}dx_{2}=\int_{R^{2}}\frac{{\tilde{I}}_{3}}{f^{5}}dx_{1}dx_{2}. \end{array}$$

Using Lemma 7 to all monomials of $R_{j}\;\left(1\leq j\leq 136\right)$, we obtain ${\bar{R}}_{j}$ which are quadratic forms in M. Doing Gaussian elimination to ${\bar{R}}_{j}\;\left(1\leq j\leq 136\right)$ to eliminate the linearly dependent ones, we obtain 48 constraints ${\tilde{R}}_{j}\;\left(1\leq j\leq 48\right)$ which are given in Appendix B.

The variables in M satisfy certain relations, such as $M_{5}M_{8}=f^{2}f_{2,0}f_{1,1}f_{1,0}f_{0,1}=M_{6}M_{7}$, which are called *intrinsic constraints*. We have 15 intrinsic constraints ${\tilde{R}}_{i}\left(49\leq i\leq 63\right)$. In total, we have 63 sixth-order constraints which are quadratic forms in M:

(25) $$\begin{array}{cc} {\tilde{R}}_{i}\;\left(i=1,\ldots ,48\right) & \\ {\tilde{R}}_{49}=M_{5}M_{8}-M_{6}M_{7},\; & {\tilde{R}}_{50}=M_{5}M_{10}-M_{6}M_{9},\\ {\tilde{R}}_{51}=M_{5}M_{12}-M_{6}M_{11},\; & {\tilde{R}}_{52}=M_{5}M_{13}-M_{6}M_{12},\\ {\tilde{R}}_{53}=M_{5}M_{14}-M_{6}M_{13},\; & {\tilde{R}}_{54}=M_{7}M_{10}-M_{8}M_{9},\\ {\tilde{R}}_{55}=M_{7}M_{12}-M_{8}M_{11},\; & {\tilde{R}}_{56}=M_{7}M_{13}-M_{8}M_{12},\\ {\tilde{R}}_{57}=M_{7}M_{14}-M_{8}M_{13},\; & {\tilde{R}}_{58}=M_{9}M_{12}-M_{10}M_{11},\\ {\tilde{R}}_{59}=M_{9}M_{13}-M_{10}M_{12},\; & {\tilde{R}}_{60}=M_{9}M_{14}-M_{10}M_{13},\\ {\tilde{R}}_{61}=M_{11}M_{13}-M_{12}^{2},\; & {\tilde{R}}_{62}=M_{11}M_{14}-M_{12}M_{13},\\ {\tilde{R}}_{63}=M_{12}M_{14}-M_{13}^{2}.\; \end{array}$$

where ${\tilde{R}}_{i}\;\left(i=1,\ldots ,48\right)$ are given in Appendix B.

The following lemma summarizes all the constraints needed in the proof.

**Lemma** **8.** *From Equations (19), (21) and (25), we obtain the following fourth-order constraints and sixth-order constraints*

(26) $$\begin{array}{c} \begin{array}{c} \int_{R^{2}}\frac{f_{1,0}{\tilde{P}}_{i}}{f^{3}}dx_{1}dx_{2}=\int_{R^{2}}\frac{f_{0,1}{\tilde{Q}}_{i}}{f^{3}}dx_{1}dx_{2}=0,i=1,\ldots ,4,\\ \int_{R^{2}}\frac{{\tilde{R}}_{j}}{f^{5}}dx_{1}dx_{2}=0,j=1,\ldots ,63, \end{array} \end{array}$$

*where ${\tilde{R}}_{j}$ are quadratic forms in M and ${\tilde{P}}_{i},{\tilde{Q}}_{i}$ are linear forms in M.*

**Proof.**  We need only to consider the equalities for ${\tilde{R}}_{j}\;\left(1\leq j\leq 48\right)$. ${\bar{R}}_{i}$ is obtained from $R_{i}$ by applying Lemma 7 to each monomial of $R_{i}$. Then by Equation (19) and Lemma 7, we have $\int_{R^{2}}\frac{R_{j}}{f^{5}}dx_{1}dx_{2}=\int_{R^{2}}\frac{{\bar{R}}_{j}}{f^{5}}dx_{1}dx_{2}=0,j=1,\ldots ,136$. ${\tilde{R}}_{j}$ are obtained from ${\bar{R}}_{j}\;\left(1\leq j\leq 136\right)$ by doing Gaussian elimination, so the ${\tilde{R}}_{j}$ are linear combinations of ${\bar{R}}_{j}$ over Q. Thus $\int_{R^{2}}\frac{{\tilde{R}}_{j}}{f^{5}}dx_{1}dx_{2}=0,j=1,\ldots ,48$. The lemma is proven.    □

### 3.4. Reduction to Semidefinite Positiveness of a Quadratic Form

In this section, we give an Θ, which is a quadratic form in M, such that Theorem 1 is true if Θ≥0, that is, Θ is a semidefinite positive polynomial when $f_{a,b}$ are treated as independent variables.

In the following key lemma, we introduce ${\tilde{J}}_{3}$ in order to generate a common factor $I=\int_{R^{2}}\frac{I_{1}}{f}dx_{1}dx_{2}$ in the proof of Lemma 10.

**Lemma** **9.** *Let*

(27) $$\begin{array}{c} \tilde{J_{3}}:={\left({\tilde{F}}_{1,0}+\tilde{P}\right)}^{2}+{\left({\tilde{F}}_{0,1}+\tilde{Q}\right)}^{2}+\tilde{R},\\ \tilde{P}:=\sum_{i=1}^{4}\alpha_{i}\tilde{P_{i}},\tilde{Q}:=\sum_{i=1}^{4}\beta_{i}\tilde{Q_{i}},\tilde{R}:=\sum_{j=1}^{63}\gamma_{j}\tilde{R_{j}},\;\alpha_{i},\beta_{i},\gamma_{j}\in R, \end{array}$$

*where ${\tilde{F}}_{1,0},{\tilde{F}}_{0,1},{\tilde{P}}_{i},{\tilde{Q}}_{i}$ are defined in Equation (21) and ${\tilde{R}}_{j}$ are defined in Equation (25). Then, $\tilde{J_{3}}$ is a quadratic form in M and satisfies*

(28) $${\left(\int_{R^{2}}\frac{I_{2}}{f^{3}}dx_{1}dx_{2}\right)}^{2}\leq \int_{R^{2}}\frac{I_{1}}{f}dx_{1}dx_{2}\int_{R^{2}}\frac{\tilde{J_{3}}}{f^{5}}dx_{1}dx_{2},$$

*where $I_{1}$ and $I_{2}$ are defined in Equation (10) and Equation (11), respectively.*

**Proof.**  $\tilde{J_{3}}$ is clearly a quadratic form in M. From Equations (10) and (11), $I_{1}=f_{1,0}^{2}+f_{0,1}^{2}$ and $I_{2}=f_{1,0}{\tilde{F}}_{1,0}+f_{0,1}{\tilde{F}}_{0,1}$ $\left(F_{1,0}={\tilde{F}}_{1,0},F_{0,1}={\tilde{F}}_{0,1}\right)$. Using the inequality (14) with $f_{1}=\frac{f_{1,0}}{\sqrt{f}}$, $f_{2}=\frac{f_{0,1}}{\sqrt{f}}$, $g_{1}=\frac{{\tilde{F}}_{0,1}}{f^{2}\sqrt{f}}$, $g_{2}=\frac{{\tilde{F}}_{1,0}}{f^{2}\sqrt{f}}$, we have

$$\begin{array}{ccc} \; & \; & {\left(\int_{R^{2}}\frac{I_{2}}{f^{3}}dx_{1}dx_{2}\right)}^{2}\\ \; & \overset{\left(27),(26\right)}{=} & {\left(\int_{R^{2}}\frac{I_{2}+f_{1,0}\tilde{P}+f_{0,1}\tilde{Q}}{f^{3}}dx_{1}dx_{2}\right)}^{2}\\ \; & \overset{\left(11\right)}{=} & {\left(\int_{R^{2}}\frac{f_{1,0}\left({\tilde{F}}_{1,0}+\tilde{P}\right)+f_{0,1}\left({\tilde{F}}_{0,1}+\tilde{Q}\right)}{f^{3}}dx_{1}dx_{2}\right)}^{2}\\ \; & \overset{\left(14\right)}{\leq } & \int_{R^{2}}\frac{f_{1,0}^{2}+f_{0,1}^{2}}{f}dx_{1}dx_{2}\int_{R^{2}}\frac{{\left({\tilde{F}}_{1,0}+\tilde{P}\right)}^{2}+{\left({\tilde{F}}_{0,1}+\tilde{Q}\right)}^{2}}{f^{5}}dx_{1}dx_{2}\\ \; & \overset{\left(26\right)}{=} & \int_{R^{2}}\frac{f_{1,0}^{2}+f_{0,1}^{2}}{f}dx_{1}dx_{2}\int_{R^{2}}\frac{{\left({\tilde{F}}_{1,0}+\tilde{P}\right)}^{2}+{\left({\tilde{F}}_{0,1}+\tilde{Q}\right)}^{2}+\tilde{R}}{f^{5}}dx_{1}dx_{2}\\ \; & \overset{\left(10),(27\right)}{=} & \int_{R^{2}}\frac{I_{1}}{f}dx_{1}dx_{2}\int_{R^{2}}\frac{{\tilde{J}}_{3}}{f^{5}}dx_{1}dx_{2}. \end{array}$$

The lemma is proven.    □

In Lemma 10, proof of Theorem 1 is finally reduced to the proof of an inequality for a quadratic form with undetermined coefficients.

**Lemma** **10.** *Let ${\tilde{I}}_{3}$ be defined in Equation (23) and ${\tilde{J}}_{3}$ be defined in Equation (27). Then Theorem 1 is true if there exist $\alpha_{i},\beta_{i},\gamma_{j}\in R$ such that*

(29) $$\Theta :=2{\tilde{I}}_{3}-{\tilde{J}}_{3}\geq 0,$$

*where Θ is a quadratic form in M.*

**Proof.**  Θ is clearly a quadratic form in M, since ${\tilde{I}}_{3}$ and ${\tilde{J}}_{3}$ are. By Lemma 3, we have

$$\begin{array}{ccc} \; & \; & 2I\left(t\right)\frac{d^{2}}{dt^{2}}I\left(t\right)-{\left(\frac{d}{dt}I\left(t\right)\right)}^{2}\\ \; & = & 2\left(\int_{R^{2}}\frac{I_{1}}{f}dx_{1}dx_{2}\right)\left(\int_{R^{2}}\frac{I_{3}}{f^{5}}dx_{1}dx_{2}\right)-{\left(\int_{R^{2}}\frac{I_{2}}{f^{3}}dx_{1}dx_{2}\right)}^{2}\\ \; & \overset{\left(28\right)}{\geq } & 2\left(\int_{R^{2}}\frac{I_{1}}{f}dx_{1}dx_{2}\right)\left(\int_{R^{2}}\frac{I_{3}}{f^{5}}dx_{1}dx_{2}\right)-\left(\int_{R^{2}}\frac{I_{1}}{f}dx_{1}dx_{2}\right)\left(\int_{R^{2}}\frac{{\tilde{J}}_{3}}{f^{5}}dx_{1}dx_{2}\right)\\ \; & \overset{\left(24\right)}{=} & \left(\int_{R^{2}}\frac{I_{1}}{f}dx_{1}dx_{2}\right)\left(\int_{R^{2}}\frac{2{\tilde{I}}_{3}-{\tilde{J}}_{3}}{f^{5}}dx_{1}dx_{2}\right)\\ \; & \overset{\left(29\right)}{\geq } & 0. \end{array}$$

Since f>0 and $I_{1}>0$, by Lemma 1, Theorem 1 is true if Θ≥0.    □

### 3.5. Prove Theorem 1 by Solving an SDP Problem

In this section, we will give an Θ in Equation (29) satisfying Θ≥0 and hence proving Theorem 1. By Lemma 10, in order to prove Theorem 1, it suffices to solve the following problem.

**Problem** **1.** *Find $\alpha_{i},\beta_{i},\gamma_{j}\in R$ such that*

(30) $$\Theta =2{\tilde{I}}_{3}-{\tilde{J}}_{3}=2{\tilde{I}}_{3}-\sum_{j=1}^{63}\gamma_{j}{\tilde{R}}_{j}-{\left({\tilde{F}}_{1,0}+\sum_{i=1}^{4}\alpha_{i}{\tilde{P}}_{i}\right)}^{2}-{\left({\tilde{F}}_{0,1}+\sum_{i=1}^{4}\beta_{i}{\tilde{Q}}_{i}\right)}^{2}\geq 0,$$

*where ${\tilde{I}}_{3}$ is defined in Equation (23); ${\tilde{R}}_{j}$ are defined in Equation (25); and ${\tilde{F}}_{1,0},{\tilde{F}}_{0,1},{\tilde{P}}_{i},{\tilde{Q}}_{i}$ are defined in Equation (21).*

It is impossible to compute $\alpha_{i},\beta_{i},\gamma_{j}$ in Problem 1 with SDP directly, since Θ is not linear in $\alpha_{i},\beta_{i}$. We use the following strategy to solve Problem 1:

S1 Expanding the squares ${\left({\tilde{F}}_{1,0}+\sum_{i=1}^{4}\alpha_{i}{\tilde{P}}_{i}\right)}^{2}$ and ${\left({\tilde{F}}_{0,1}+\sum_{i=1}^{4}\beta_{i}{\tilde{Q}}_{i}\right)}^{2}$ and deleting the terms $-{\left(\sum_{i=1}^{4}\alpha_{i}{\tilde{P}}_{i}\right)}^{2}$ and $-{\left(\sum_{i=1}^{4}\beta_{i}{\tilde{Q}}_{i}\right)}^{2}$, we obtain Problem 2 which is weaker than Problem 1.
S2 Since $\tilde{\Theta }$ in Problem 2 is linear in $\alpha_{i},\beta_{i},\gamma_{j}$, we can use SDP to solve Problem 2 and let ${\tilde{\alpha }}_{i},{\tilde{\beta }}_{i},{\tilde{\gamma }}_{j}$ be the solutions.
S3 Let $\Theta_{1}$ be obtained from Θ by substituting $\alpha_{i},\beta_{i}$ with ${\tilde{\alpha }}_{i},{\tilde{\beta }}_{i}$. Then, $\Theta_{1}$ is linear in $\gamma_{j}$ and we can use SDP to compute $\gamma_{j}$ such that $\Theta_{1}$≥0 is true. Under this condition, Problem 1 becomes Problem 5, and it suffices to solve Problem 5 in order to prove Theorem 1.

**Problem** **2.** *Find $\alpha_{i},\beta_{i},\gamma_{j}\in R$ such that*

$$\tilde{\Theta }:=\left(2{\tilde{I}}_{3}-{\tilde{F}}_{1,0}^{2}-{\tilde{F}}_{0,1}^{2}\right)-\sum_{j=1}^{63}\gamma_{j}{\tilde{R}}_{j}-2\sum_{i=1}^{4}\alpha_{i}{\tilde{F}}_{1,0}{\tilde{P}}_{i}-2\sum_{i=1}^{4}\beta_{i}{\tilde{F}}_{0,1}{\tilde{Q}}_{i}\geq 0,$$

*where ${\tilde{I}}_{3}$ is defined in Equation (23); ${\tilde{R}}_{j}$ are defined in Equation (25); and ${\tilde{F}}_{1,0},{\tilde{F}}_{0,1},{\tilde{P}}_{i},{\tilde{Q}}_{i}$ are defined in Equation (21).*

Since $\tilde{\Theta }$ is a quadratic form in M, it is well known that $\tilde{\Theta }$≥0 is equivalent to the fact that the symmetric matrix $\hat{\Theta }$$\in R^{14\times 14}$ of $\tilde{\Theta }$ is *positive semidefinite*, that is, $\hat{\Theta }$⪰0 [19]. In other words, Problem 2 is equivalent to the following SDP problem [19].

**Problem** **3.**
$$\begin{array}{c} \min_{\alpha_{i},\beta_{i},\gamma_{j}\in R}\;1\;s.t.\\ \hat{\Theta }:=\left(2{\hat{I}}_{3}-\hat{F_{1,0}^{2}}-\hat{F_{0,1}^{2}}\right)-\sum_{j=1}^{63}\gamma_{j}{\hat{R}}_{j}-\sum_{i=1}^{4}2\alpha_{i}\hat{F_{1,0}P_{i}}-\sum_{i=1}^{4}2\beta_{i}\hat{F_{0,1}Q_{i}}⪰0, \end{array}$$

*where $\hat{Q}\in R^{n\times n}$ is the corresponding symmetric matrix for any quadratic form Q in M and n=|M|=14.*

We set the objective function to be 1, which means that it suffices to satisfy the constraints.

We actually solve the following dual problem [19] of Problem 3:

**Problem** **4.**
$$\begin{array}{cccc} \; & \max_{X} & \; & -\mathrm{trace}(X^{T}\hat{I})\\ \; & s.t. & \; & \mathrm{trace}(X^{T}{\hat{R}}_{j})=0,j=1,\ldots ,63\\ \; & & \; & \mathrm{trace}(X^{T}2\hat{F_{1,0}P_{i}})=0,i=1,\ldots ,4\\ \; & & \; & \mathrm{trace}(X^{T}2\hat{F_{0,1}Q_{i}})=0,i=1,\ldots ,4\\ \; & & \; & X⪰0 \end{array}$$

*where $\hat{I}:=2{\hat{I}}_{3}-\hat{F_{1,0}^{2}}-\hat{F_{0,1}^{2}},X\in R^{n\times n}$, and n=|M|=14.*

**Remark** **1.** *If not using differential forms to reduce the polynomials into quadratic forms in M, then we need to consider all differential monomials with total degree 3 and total order ≤6 as the bases for the SDP Problem 4. In such a case, n=100 instead of n=14, and we need to solve a much larger SDP problem for $X\in R^{n\times n}$.*

We use the CVX package in Matlab [26] to solve Problem 4. The program is given in Appendix A. Our complete code and data are available (accessed on 30 November 2022) at https://github.com/liujunliang19/sqrt-convex.

With CVX, we obtain a set of solutions for $\gamma_{j},\alpha_{i},\beta_{i}$, which are given in Appendix C. From the above discussions, we see that these values are also solutions to Problem 2.

Finally, according to step **S3** just above Problem 2, we put the solutions for $\alpha_{i},\beta_{i}$ back into Θ in Problem 1 and obtain the following problem.

**Problem** **5.** *Find $\lambda_{j}\in R$ such that*

(31) $$\Theta_{1}:=2{\tilde{I}}_{3}-\sum_{j=1}^{63}\lambda_{j}{\tilde{R}}_{j}-{\left({\tilde{F}}_{1,0}-\frac{29}{110}{\tilde{P}}_{1}-\frac{32}{139}{\tilde{P}}_{4}\right)}^{2}-{\left({\tilde{F}}_{0,1}-\frac{29}{110}{\tilde{Q}}_{2}-\frac{23}{100}{\tilde{Q}}_{3}\right)}^{2}\geq 0,$$

*where ${\tilde{I}}_{3}$ is defined in Equation (23); ${\tilde{R}}_{j}$ are defined in Equation (25); and ${\tilde{F}}_{1,0},{\tilde{F}}_{0,1},{\tilde{P}}_{i},{\tilde{Q}}_{i}$ are defined in Equation (21).*

Similar to Problems 3 and 4, we obtain a set of solutions for $\lambda_{j}$, which are given in Appendix D. Now $\Theta_{1}$ is a semi-positive quadratic form and it is well known that $\Theta_{1}$ can be written as an SOS. The value of $\Theta_{1}$ as well as its SOS representation are given in Appendix E. Hence, we solve Problem 1 and therefore prove Theorem 1.

**Remark** **2.** *Note that the SOS given in Appendix E provides an explicit and direct proof for Theorem 1 and the solution procedure for the SDP is not needed, similar to Equation (17) for the case of n=1. Of course, the SOS in Appendix E is quite large and difficult to check manually. In order for interested readers to check the proof with a mathematical software system, we also give the complete code and data in https://github.com/liujunliang19/sqrt-convex (accessed on 30 November 2022). The SOS expression for $H_{1}$ is in the bottom of our Maple code named* sqrt-convex2.mw*, which can be run directly.*

**Remark** **3.** *We also try to use the above approach to prove the log-convexity of the Fisher information along heat flow for n=2. The CVX program returns failed. Thus, we cannot prove the log-convexity with the above approach. We also cannot say that the log-convexity is not correct, since the log-convexity is not equivalent to Problem 3.*

**Remark** **4.** *Theorem 1 is stronger than the CMC for the third-order derivative with dimension two. In other words, given Theorem 1, we can obtain $\frac{d^{3}}{dt^{3}}H\left(X_{t}\right)\geq 0\;\left(n=2\right)$. Using Lemma 1, we obtain $2I\left(t\right)\frac{d^{2}}{dt^{2}}I\left(t\right)\geq {\left(\frac{d}{dt}I\left(t\right)\right)}^{2}\geq 0\;\left(n=2\right)$. Since I(t)≥0, we have $\frac{d^{2}}{dt^{2}}I\left(t\right)\geq 0\;\left(n=2\right)$. Using Equation (3), we have $\frac{d^{3}}{dt^{3}}H\left(X_{t}\right)=\frac{1}{2}\frac{d^{2}}{dt^{2}}I\left(t\right)\geq 0\;\left(n=2\right)$.*

## 4. Conclusions

In this paper, we prove the sqrt-convexity of Fisher Information along heat flow in dimension two. It is easy to find that this conclusion is weaker than the log-convexity conjecture. However, it is stronger than the CMC for the third-order derivative with dimension two.

The proof is based on the SDP method. In order to reduce the size of the SDP problem, we prove that any sixth-order differential form can be reduced to an “equivalent” differential polynomial which is a quadratic form in certain new variables. Based on this fact, we reduce the sixth-order differential forms into quadratic forms in a set of new variables, which reduces the size of the SDP problem significantly.

For possible future research directions, it is interesting to prove the sqrt-convexity for higher dimensions (n≥3) using the method given in this paper. In this case, the main difficulty is to establish inequality (27) in higher dimensions. Another question is to prove the log-convexity by introducing more constraints or new methods to solve Problem 1 without using the relaxation method used in Problem 2. The methods introduced in this paper may be used to prove other EPI inequalities related with the heat equations.

## Data Availability

The code for the SDP solver and data are available (accessed on 30 November 2022) at https://github.com/liujunliang19/sqrt-convex.

## Appendix A. Matlab Codes for SDP

The following Matlab codes are used to solve Problem 4.

cvx_begin
variable Z(n,n) symmetric
dual variable y
maximize(**trace**(C∗X))
subject to
[**trace**(A_1∗Z),**trace**(A_2∗Z),...,**trace**(A_m∗Z)]’==
**zeros**(m,1):y;
Z == semidefinite(n);
cvx_end

## Appendix B

We give ${\tilde{R}}_{i}\;\left(1\leq i\leq 48\right)$ in Equation (25).

$$\begin{array}{cc} \; & {\tilde{R}}_{1}=4M_{5}M_{12}-\frac{16}{5}M_{11}M_{12}\\ \; & {\tilde{R}}_{2}=M_{8}M_{14}-\frac{4}{5}M_{13}M_{14}\\ \; & {\tilde{R}}_{3}=M_{1}M_{11}+3M_{5}^{2}-\frac{12}{5}M_{11}^{2}\\ \; & {\tilde{R}}_{4}=M_{2}M_{11}+3M_{5}M_{7}-\frac{12}{5}M_{11}M_{12}\\ \; & {\tilde{R}}_{5}=M_{3}M_{14}+3M_{8}M_{10}-\frac{12}{5}M_{13}M_{14}\\ \; & {\tilde{R}}_{6}=M_{4}M_{14}+3M_{10}^{2}-\frac{12}{5}M_{14}^{2}\\ \; & {\tilde{R}}_{7}=3M_{5}M_{13}-\frac{1}{2}M_{9}M_{11}-2M_{12}^{2}\\ \; & {\tilde{R}}_{8}=M_{1}M_{12}+2M_{5}M_{6}+M_{5}M_{7}-\frac{12}{5}M_{11}M_{12}\\ \; & {\tilde{R}}_{9}=M_{2}M_{14}+3M_{8}^{2}+\frac{9}{2}M_{9}M_{13}-6M_{13}^{2}\\ \; & {\tilde{R}}_{10}=M_{3}M_{11}+3M_{7}^{2}+\frac{3}{4}M_{9}M_{11}-3M_{12}^{2}\\ \; & {\tilde{R}}_{11}=M_{4}M_{13}+M_{8}M_{10}+2M_{9}M_{10}-\frac{12}{5}M_{13}M_{14}\\ \; & {\tilde{R}}_{12}=-M_{5}M_{9}+M_{7}^{2}+\frac{5}{4}M_{9}M_{11}-M_{12}^{2}\\ \; & {\tilde{R}}_{13}=-3M_{6}M_{10}+3M_{8}^{2}+\frac{45}{2}M_{9}M_{13}-18M_{13}^{2}\\ \; & {\tilde{R}}_{14}=M_{1}M_{13}+2M_{5}M_{8}+M_{6}^{2}-\frac{1}{2}M_{9}M_{11}-2M_{12}^{2}\\ \; & {\tilde{R}}_{15}=M_{2}M_{12}+2M_{5}M_{8}+M_{7}^{2}+\frac{3}{4}M_{9}M_{11}-3M_{12}^{2}\\ \; & {\tilde{R}}_{16}=M_{3}M_{13}+2M_{7}M_{10}+M_{8}^{2}+\frac{9}{2}M_{9}M_{13}-6M_{13}^{2}\\ \; & {\tilde{R}}_{17}=2M_{2}M_{7}-2M_{3}M_{5}+4M_{5}M_{8}-6M_{5}M_{9}+2M_{7}^{2}+\frac{15}{2}M_{9}M_{11}-6M_{12}^{2}\\ \; & {\tilde{R}}_{18}=\frac{2}{3}M_{3}M_{7}-\frac{2}{3}M_{4}M_{5}-\frac{8}{3}M_{5}M_{10}+4M_{7}M_{8}-\frac{4}{3}M_{7}M_{9}+10M_{9}M_{12}-8M_{11}M_{14}\\ \; & {\tilde{R}}_{19}=M_{1}M_{8}+M_{1}M_{9}-M_{2}M_{6}-M_{2}M_{7}+2M_{6}^{2}-2M_{7}^{2}-\frac{5}{2}M_{9}M_{11}+2M_{12}^{2}\\ \; & {\tilde{R}}_{20}=-M_{2}M_{8}+\frac{1}{3}M_{3}M_{7}+\frac{2}{3}M_{4}M_{5}+\frac{8}{3}M_{5}M_{10}-4M_{7}M_{8}+\frac{4}{3}M_{7}M_{9}-10M_{9}M_{12}+8M_{11}M_{14}\\ \; & {\tilde{R}}_{21}=\frac{12}{5}M_{3}M_{9}-\frac{8}{5}M_{4}M_{6}-\frac{4}{5}M_{4}M_{7}+\frac{12}{5}M_{5}M_{9}+\frac{24}{5}M_{6}M_{10}-\frac{12}{5}M_{7}^{2}-\frac{8}{5}M_{7}M_{10}-\frac{16}{5}M_{9}^{2}\\ \; & -3M_{9}M_{11}-12M_{9}M_{13}+\frac{12}{5}M_{12}^{2}+\frac{48}{5}M_{13}^{2}\\ \; & {\tilde{R}}_{22}=-\frac{5}{3}M_{3}M_{8}+\frac{4}{3}M_{3}M_{9}-\frac{1}{3}M_{4}M_{6}+\frac{2}{3}M_{4}M_{7}+\frac{4}{3}M_{5}M_{9}+\frac{8}{3}M_{6}M_{10}-\frac{4}{3}M_{7}^{2}-2M_{7}M_{10}\\ & -\frac{2}{3}M_{9}^{2}-\frac{5}{3}M_{9}M_{11}-15M_{9}M_{13}+\frac{4}{3}M_{12}^{2}+12M_{13}^{2}\\ \; & {\tilde{R}}_{23}=3M_{1}M_{9}-6M_{2}M_{6}+3M_{3}M_{5}+5M_{3}M_{8}-4M_{3}M_{9}+M_{4}M_{6}-2M_{4}M_{7}-4M_{5}M_{9}+6M_{6}^{2}\\ \; & -8M_{6}M_{10}-2M_{7}^{2}+6M_{7}M_{10}+2M_{9}^{2}-\frac{5}{2}M_{9}M_{11}+45M_{9}M_{13}+2M_{12}^{2}-36M_{13}^{2}\\ \; & {\tilde{R}}_{24}=5M_{5}M_{11}-4M_{11}^{2}\\ \; & {\tilde{R}}_{25}=5M_{10}M_{14}-4M_{14}^{2}\\ \; & {\tilde{R}}_{26}=5M_{7}M_{11}-4M_{11}M_{12}\\ \; & {\tilde{R}}_{27}=-20M_{9}M_{14}+16M_{13}M_{14}\\ \; & {\tilde{R}}_{28}=M_{1}M_{6}-M_{2}M_{5}\\ \; & {\tilde{R}}_{29}=2M_{5}M_{14}-2M_{9}M_{12}\\ \; & {\tilde{R}}_{30}=M_{6}M_{14}-6M_{9}M_{13}+4M_{13}^{2}\\ \; & {\tilde{R}}_{31}=4M_{7}M_{12}+M_{9}M_{11}-4M_{12}^{2}\\ \; & {\tilde{R}}_{32}=2M_{7}M_{14}+3M_{9}M_{13}-4M_{13}^{2}\\ \; & {\tilde{R}}_{33}=M_{4}M_{11}+3M_{7}M_{9}-3M_{9}M_{12}\\ \; & {\tilde{R}}_{34}=M_{1}M_{14}+3M_{6}M_{8}-3M_{9}M_{12}\\ \; & {\tilde{R}}_{35}=3M_{7}M_{13}+2M_{9}M_{12}-4M_{11}M_{14}\\ \; & {\tilde{R}}_{36}=-M_{4}M_{8}+M_{4}M_{9}+2M_{8}M_{10}-2M_{9}M_{10}\\ \; & {\tilde{R}}_{37}=-M_{1}M_{3}+M_{1}M_{8}+M_{2}^{2}-M_{2}M_{7}\\ \; & {\tilde{R}}_{38}=2M_{1}M_{7}-2M_{2}M_{5}+4M_{5}M_{6}-4M_{5}M_{7}\\ \; & {\tilde{R}}_{39}=2M_{3}M_{10}-2M_{4}M_{8}+4M_{8}M_{10}-4M_{9}M_{10}\\ \; & {\tilde{R}}_{40}=M_{4}M_{12}+2M_{7}M_{10}+M_{9}^{2}-3M_{9}M_{13}\\ \; & {\tilde{R}}_{41}=12M_{5}M_{10}-12M_{7}M_{8}-30M_{9}M_{12}+24M_{11}M_{14}\\ \; & {\tilde{R}}_{42}=M_{3}M_{12}+2M_{7}M_{8}+M_{7}M_{9}+2M_{9}M_{12}-4M_{11}M_{14}\;\;\;\;\;\;\;\;\;\;\;\;\;\;\;\;\;\;\;\;\;\;\;\;\;\;\;\;\;\;\;\;\;\;\;\;\;\;\;\;\;\;\;\;\;\;\;\;\;\;\;\;\;\;\;\;\;\;\;\;\;\;\;\;\;\;\\ \; & {\tilde{R}}_{43}=M_{2}M_{13}+M_{6}M_{8}+2M_{7}M_{8}+2M_{9}M_{12}-4M_{11}M_{14}\\ \; & {\tilde{R}}_{44}=2M_{2}M_{10}-2M_{3}M_{8}+4M_{6}M_{10}-4M_{7}M_{10}-30M_{9}M_{13}+24M_{13}^{2}\\ \; & {\tilde{R}}_{45}=3M_{1}M_{10}-2M_{3}M_{7}-M_{4}M_{5}-4M_{5}M_{10}+6M_{6}M_{8}-2M_{7}M_{9}\\ \; & {\tilde{R}}_{46}=M_{2}M_{4}-M_{3}^{2}+2M_{3}M_{8}+M_{3}M_{9}-M_{4}M_{6}-2M_{4}M_{7}+2M_{6}M_{10}-2M_{9}^{2}\\ \; & {\tilde{R}}_{47}=-M_{2}M_{9}+M_{3}M_{6}+4M_{5}M_{10}-2M_{6}M_{8}-4M_{7}M_{8}+2M_{7}M_{9}-10M_{9}M_{12}+8M_{11}M_{14}\\ \; & {\tilde{R}}_{48}=-M_{1}M_{4}+3M_{1}M_{10}+M_{2}M_{3}-M_{2}M_{8}-M_{2}M_{9}-M_{3}M_{7}+4M_{6}M_{8}-4M_{7}M_{8}\\ \; & -10M_{9}M_{12}\;+8M_{11}M_{14} \end{array}$$

## Appendix C. Solutions to Problem 2

We give the solutions $\alpha_{i}\;\left(1\leq i\leq 4\right)$, $\beta_{i}\;\left(1\leq i\leq 4\right)$, $\gamma_{j}\;\left(1\leq j\leq 63\right)$ to Problem 4, which are also solutions to Problems 2 and 3.

$$\begin{array}{cccccccccc} \; & \alpha_{1}=-29/110 & \; & \alpha_{2}=0 & \; & \alpha_{3}=0 & \; & \alpha_{4}=-32/139\\ \; & \beta_{1}=0 & \; & \beta_{2}=-29/110 & \; & \beta_{3}=-23/100 & \; & \beta_{4}=0\\ \; & \gamma_{1}=0 & \; & \gamma_{2}=0 & \; & \gamma_{3}=-283/207 & \; & \gamma_{4}=0 & \; & \gamma_{5}=0\\ \; & \gamma_{6}=-175/128 & \; & \gamma_{7}=93/130 & \; & \gamma_{8}=0 & \; & \gamma_{9}=-85/92 & \; & \gamma_{10}=-110/119\\ \; & \gamma_{11}=0 & \; & \gamma_{12}=-114/83 & \; & \gamma_{13}=779/161 & \; & \gamma_{14}=-76/83\; & \; & \gamma_{15}=-493/219\\ \; & \gamma_{16}=-232/103 & \; & \gamma_{17}=270/173 & \; & \gamma_{18}=0 & \; & \gamma_{19}=-167/68 & \; & \gamma_{20}=0\\ \; & \gamma_{21}=297/52 & \; & \gamma_{22}=-107/188 & \; & \gamma_{23}=409/256 & \; & \gamma_{24}=37/113\; & \; & \gamma_{25}=37/113\\ \; & \gamma_{26}=0 & \; & \gamma_{27}=0 & \; & \gamma_{28}=0 & \; & \gamma_{29}=0 & \; & \gamma_{30}=24/85\\ \; & \gamma_{31}=69/112 & \; & \gamma_{32}=85/69 & \; & \gamma_{33}=0 & \; & \gamma_{34}=0 & \; & \gamma_{35}=0\\ \; & \gamma_{36}=0 & \; & \gamma_{37}=481/285 & \; & \gamma_{38}=0 & \; & \gamma_{39}=0 & \; & \gamma_{40}=-118/129\\ \; & \gamma_{41}=0 & \; & \gamma_{42}=0 & \; & \gamma_{43}=0 & \; & \gamma_{44}=127/152 & \; & \gamma_{45}=0\\ \; & \gamma_{46}=-27/16 & \; & \gamma_{47}=0 & \; & \gamma_{48}=0 & \; & \gamma_{49}=118/101 & \; & \gamma_{50}=0\\ \; & \gamma_{51}=0 & \; & \gamma_{52}=-555/247 & \; & \gamma_{53}=0 & \; & \gamma_{54}=256/219 & \; & \gamma_{55}=14/51\\ \; & \gamma_{56}=0 & \; & \gamma_{57}=-241/88 & \; & \gamma_{58}=0 & \; & \gamma_{59}=8/79 & \; & \gamma_{60}=0\\ \; & \gamma_{61}=13/29 & \; & \gamma_{62}=0 & \; & \gamma_{63}=204/455 \end{array}$$

## Appendix D. Solutions to Problem 5

We give the solutions $\lambda_{j}\;\left(1\leq j\leq 63\right)$ to Problem 5.

$$\begin{array}{cccccccccc} \; & \lambda_{1}=0 & \; & \lambda_{2}=0 & \; & \lambda_{3}=-363/248 & \; & \lambda_{4}=0 & \; & \lambda_{5}=0\\ \; & \lambda_{6}=-157/109 & \; & \lambda_{7}=47/63 & \; & \lambda_{8}=0 & \; & \lambda_{9}=-355/317\; & \; & \lambda_{10}=-208/307\\ \; & \lambda_{11}=0 & \; & \lambda_{12}=-208/99 & \; & \lambda_{13}=4372/845\; & \; & \lambda_{14}=-111/92 & \; & \lambda_{15}=-255/104\\ \; & \lambda_{16}=-529/241 & \; & \lambda_{17}=233/132 & \; & \lambda_{18}=0\; & \; & \lambda_{19}=-645/253 & \; & \lambda_{20}=0\\ \; & \lambda_{21}=821/142 & \; & \lambda_{22}=-21/68 & \; & \lambda_{23}=263/151\; & \; & \lambda_{24}=43/108 & \; & \lambda_{25}=107/283\\ \; & \lambda_{26}=0 & \; & \lambda_{27}=0 & \; & \lambda_{28}=0 & \; & \lambda_{29}=0 & \; & \lambda_{30}=227/328\\ \; & \lambda_{31}=61/76 & \; & \lambda_{32}=97/75 & \; & \lambda_{33}=0 & \; & \lambda_{34}=0 & \; & \lambda_{35}=0\\ \; & \lambda_{36}=0 & \; & \lambda_{37}=342/157 & \; & \lambda_{38}=0 & \; & \lambda_{39}=0\; & \; & \lambda_{40}=-191/186\\ \; & \lambda_{41}=0 & \; & \lambda_{42}=0 & \; & \lambda_{43}=0 & \; & \lambda_{44}=188/181 & \; & \lambda_{45}=0\\ \; & \lambda_{46}=-243/128 & \; & \lambda_{47}=0 & \; & \lambda_{48}=0 & \; & \lambda_{49}=281/522\; & \; & \lambda_{50}=0\\ \; & \lambda_{51}=0 & \; & \lambda_{52}=-269/187 & \; & \lambda_{53}=0 & \; & \lambda_{54}=97/114\; & \; & \lambda_{55}=1/36\\ \; & \lambda_{56}=0 & \; & \lambda_{57}=-307/137 & \; & \lambda_{58}=0 & \; & \lambda_{59}=-263/296\; & \; & \lambda_{60}=0\\ \; & \lambda_{61}=-10/181 & \; & \lambda_{62}=0 & \; & \lambda_{63}=-25/84 \end{array}$$

## Appendix E. SOS Expression of Θ_1_

The value of $\Theta_{1}$ is:

$\begin{array}{l} \Theta_{1}=M_{1}^{2}+\frac{28}{157}M_{1}M_{3}-\frac{78}{55}M_{1}M_{5}+\frac{7133009}{5521219}M_{1}M_{8}-\frac{6453649}{5310217}M_{1}M_{9}+\frac{5581}{13640}M_{1}M_{11}+\frac{3653}{12788}M_{1}M_{13}\\ +\frac{443}{157}M_{2}^{2}-\frac{13}{128}M_{2}M_{4}-\frac{5041031}{1910150}M_{2}M_{6}-\frac{1614857}{541650}M_{2}M_{7}+\frac{5022}{9955}M_{2}M_{10}+\frac{3983}{2600}M_{2}M_{12}\\ +\frac{1139}{17435}M_{2}M_{14}+\frac{397}{128}M_{3}^{2}+\frac{44197}{49830}M_{3}M_{5}-\frac{10036650925}{4133321792}M_{3}M_{8}-\frac{50886897819}{16213582720}M_{3}M_{9}-\frac{6366}{16885}M_{3}M_{11}\\ +\frac{42683}{33499}M_{3}M_{13}+M_{4}^{2}-\frac{602116651}{583222400}M_{4}M_{6}+\frac{419279509}{291611200}M_{4}M_{7}-\frac{78}{55}M_{4}M_{10}+\frac{497}{4650}M_{4}M_{12}\\ +\frac{2313}{5995}M_{4}M_{14}+\frac{543657}{750200}M_{5}^{2}-\frac{3509920763}{2386432620}M_{5}M_{8}+\frac{2688875063}{25080385770}M_{5}M_{9}-\frac{205631}{326700}M_{5}M_{11}\\ +\frac{96556}{248115}M_{5}M_{13}+\frac{505083713}{382030000}M_{6}^{2}-\frac{86969}{652500}M_{6}M_{7}+\frac{139595170060937}{490605224824000}M_{6}M_{10}-\frac{358527}{467500}M_{6}M_{12}\\ +\frac{35063}{451000}M_{6}M_{14}+\frac{22297627286581783}{8281308816495000}M_{7}^{2}-\frac{8095283498600389}{6438694624495375}M_{7}M_{10}-\frac{1203457}{427500}M_{7}M_{12}\\ +\frac{78722}{565125}M_{7}M_{14}+\frac{2270979774558}{1247276139265}M_{8}^{2}+\frac{393049}{2202594}M_{8}M_{9}+\frac{141277}{275220}M_{8}M_{11}-\frac{4809243}{2646977}M_{8}M_{13}\\ +\frac{158124697228757}{104796491910720}M_{9}^{2}+\frac{1386601574744315513}{4899089794897378080}M_{9}M_{11}-\frac{93944237793653606351280127}{77398522886148846455005400}M_{9}M_{13}\\ +\frac{215856}{329725}M_{10}^{2}+\frac{121743}{407000}M_{10}M_{12}-\frac{452981}{856075}M_{10}M_{14}+\frac{1021241}{5063850}M_{11}^{2}-\frac{595422}{1383745}M_{11}M_{13}\\ +\frac{254245231794897253159}{199355947139484135000}M_{12}^{2}-\frac{21653}{115500}M_{12}M_{14}+\frac{262837614282547093857383}{236176835310829086828700}M_{13}^{2}+\frac{16560133}{93312175}M_{14}^{2} \end{array}$

Next, we give the SOS expression of $\Theta_{1}=\sum_{i=1}^{14}\kappa_{i}{\left(\sum_{j=i}^{14}\mu_{i,j}M_{i}M_{j}\right)}^{2}$. The parameters not mentioned above are 0.

$\begin{array}{l} \kappa_{1}=1,\kappa_{2}=443/157,\kappa_{3}=9760565/3155072,\kappa_{4}=29005915/29032448,\\ \kappa_{5}=208680430142632541/1502617428470367000,\\ \kappa_{6}=254845206367035693279770017/616731381005248623385150000,\\ \kappa_{7}=3205317995201138587458416275628086155489003267179/\\ 3029566856759437193752730379916263883723707019560,\\ \kappa_{8}=129346265420106916568678950322309264192296788391/\\ 152823044461521957391224862681910484518198052480,\\ \kappa_{9}=27809943919324934445244380001625250511737806865364775965142311767209/\\ 122092260292579205097028684025778705457781727828878414585029349764800,\\ \kappa_{10}=424832608852609712256798704857924428777343282773304441738461658519256056247379/\\ 4925558197128944002809370841764515187045912505370123534195449522984520918307840,\\ \kappa_{11}=138872556785505338170420018992660029962225023247254592880803918765702192997559309557/\\ 17445046689968868434326878732657870806791020754286779471873299119392675650009247955712,\\ \kappa_{12}=140278968440223253899895444891338549285051104801125327426403925721337636757988731331\\ 8704835141306089/3479869695739952558003981016858935978619719181780257106754389615137471\\ 5789547779914385019009658472680,\\ \kappa_{13}=604447646634282248350351358416617870250513088153272472223118344345175656211357785467\\ 5650055082115013879911559085796418320811/1930330919766627607839400790038364046255969055\\ 75207876468599876531144568637730192598055535034357490574533326935257744858897200,\\ \kappa_{14}=573376402650759464814193517607183020227696371005835999431768228660749434037209496944\\ 3798520188034237831888760036314143950893/1096088362600344822912522877798221456332750932\\ 362094411886308854783332104852973001677722701445651310610269715521038140265139000,\\ \mu_{i,i}=1\left(1\leq i\leq 14\right),\\ \mu_{1,3}=14/157,\\ \mu_{1,5}=-39/55,\mu_{1,8}=7133009/11042438,\mu_{1,9}=-6453649/10620434,\\ \mu_{1,11}=5581/27280,\mu_{1,13}=3653/25576,\\ \mu_{2,4}=-2041/113408,\mu_{2,6}=-791441867/1692392900,\mu_{2,7}=-1614857/3056700,\\ \mu_{2,10}=394227/4410065,\mu_{2,12}=625331/2303600,\mu_{2,14}=178823/15447410,\\ \mu_{3,5}=39831683744/243184476975,\mu_{3,8}=-65560045349620353/159483119878645585,\\ \mu_{3,9}=-306382997632769383/625596768584742350,\mu_{3,11}=-31040751344/464456485525,\\ \mu_{3,13}=303544066272/1504058167901,\\ \mu_{4,6}=-18070850742068032/33437308892230375,\mu_{4,7}=6316978375269568/9119266061517375,\\ \mu_{4,10}=-203628062976/288753883825,\mu_{4,12}=4537774192/67438752375,\\ \mu_{4,14}=10676034477952/55123275954725),\\ \mu_{5,8}=-384773385951616686292970285/773914896181275444840093733,\\ \mu_{5,9}=748702786947138499895423/805251661545357453927439,\\ \mu_{5,11}=-375807353452502501303/384389352322729140522,\\ \mu_{5,13}=26671705865381497872323625/19133233141225605982754783,\\ \mu_{6,7}=-62733895020493663769897851306/66514598861796315946019974437,\\ \mu_{6,10}=-18192447632086750779448425576715/62363680140490038573879080400104,\\ \mu_{6,12}=8261208607905215485888011779/308107854497746153175241950553,\\ \mu_{6,14}=138676599045243082750813383316315/361032323039607556705731629293441,\\ \mu_{7,10}=-979758613168820041603217521125493464258092802119268806781/\\ 8564763461513580075513760193457485740528012470323034179704,\\ \mu_{7,12}=1854028485320471742919224371249503332742540456277079/\\ 1887932299173470628013007186344942745583022924368431,\\ \mu_{7,14}=9727374195894641621834927966046970684551801110074467927/\\ 100338964846699164038247636061398850749555639257552538545,\\ \mu_{8,9}=-12779618730487291463191846129496757762906461696978267/\\ 52695409839620717596246667003408149613413326996916618,\\ \mu_{8,11}=-122118378516557819068765438108425804752485918809740/\\ 3692965224009472574952352710652251801954265605351441,\\ \mu_{8,13}=-94939523121681601513656137577561549398850896305080/\\ 124043068537882532989363113359094584360412620066969,\\ \mu_{9,11}=3550893523515801884306409421029786766431528385700436202236083804607990380/\\ 25638571308047962888694144055478352577031830643123959622559965206737088489,\\ \mu_{9,13}=-7945848812909987868058927152552583529722064868069915968379981242142702296181690979\\ 8/94546593373096755745578683571472373707556639624258871883954420295939113462405459891,\\ \mu_{10,12}=63405502958698345886668393872792270425893974774905390610957167604969534529672/\\ 424832608852609712256798704857924428777343282773304441738461658519256056247379,\\ \mu_{10,14}=-905925800492569322388118625829368447231159919975109986163690065715646157190607027\\ 267176/10703799622824271570367336429542375230077925714200000663940636741048182250415120216\\ 16865,\\ \mu_{11,13}=-105476225900697044986144688198887230178243505011443868627920704994758066688450160\\ 26062203618333419498/101430268009464405995394306109144217152680242592189500043254326952447\\ 19795065794880212493780307453215,\\ \mu_{12,14}=-789055628026834299501182987960627756240418386432379047235785969484045323353728731\\ 20303046433951024275381808209/385246930821572992901155980884640884342608948610835474003000\\ 440010481668921844997018828943090780516847699842935. \end{array}$
